# Post Paddle Boarding Atrial Fibrillation in an Aging Athlete

**DOI:** 10.7759/cureus.22577

**Published:** 2022-02-24

**Authors:** Mohamed Faris, Jason Goebel, Robert Sherertz

**Affiliations:** 1 Department of Internal Medicine, Grand Strand Regional Medical Center, Myrtle Beach, USA; 2 Electrophysiology, Grand Strand Medical Center, Myrtle Beach, USA

**Keywords:** ablation, antiarrhythmics, electrophysiology, exercise, atrial fibrillation

## Abstract

Exercise-induced atrial fibrillation has been described in the literature and is a well-known phenomenon. It has been mostly described in long-distance runners. We present a case of a 69-year-old white male who had recurring atrial fibrillation with rapid ventricular response while paddle boarding, but not during other activities such as tennis and cycling. This case highlights the rationale behind different activities provoking atrial fibrillation and the need for multidisciplinary management of this entity including consulting with an electrophysiologist for possible early ablation.

## Introduction

Age is the dominant risk factor for new-onset atrial fibrillation (AF). In one cross-sectional study of almost 2 million subjects in a health maintenance organization in the United States, the overall prevalence of AF was 1 percent; 70 percent were at least 65 years old and about half were ≥ 75 years old [1]. In contrast, there is uncertainty as to whether exercise is a definitive risk factor for developing AF. In this report, we discuss a 69-year-old male physician who developed AF, first transiently, at the age of 55 after a large, postoperative pulmonary embolus, and not again until age 69, when it occurred repetitively with paddle boarding, but not after other forms of exercise or at any other time. We will review the patient’s exercise and medical history and then review the post-exercise AF medical literature as it pertains to this patient.

## Case presentation

The patient’s first episode of AF occurred in 2005 after being hospitalized for idiopathic small bowel obstruction requiring abdominal exploration and subsequently complicated by deep venous thrombosis/pulmonary infarction. In 2014, the patient had an episode of transient global amnesia associated with paddle boarding. At that time, no evidence of cerebrovascular accident or ongoing AF was noted. In 2019, the patient developed AF during paddle boarding. His only other past medical history was mild hypercholesterolemia. There was no history of illicit or performance-enhancing drugs or thyroid abnormalities.

The patient was a participant in organized sports during early childhood (baseball and wrestling), and long-term thereafter (volleyball: high school through the early 40s, ice hockey: mid-40s, tennis: high school until the early 50s, and a cervical disk rupture). In addition to organized sports, he regularly exercised from middle school onwards, scoring 470/500 on the President’s fitness program in 9th grade, and running a 4:30 mile his senior year of high school. At the age of 28, he ran a 66-minute ten-mile marathon. From age thirty until now, he has done some kind of aerobic exercise 3-5 times weekly, first running, then Nordic track, then indoor cycling on a spinner bike standing up (30-60 minutes, 3 times per week), and going up and downstairs with a 45 lb. backpack (30 minutes each session). His most recent aerobic sport was stand up paddle boarding, which started in 2015 and was done year-round (40-60 minutes per day, 2-5 times per week) until October 2019, when it was stopped due to AF.

**Figure 1:**
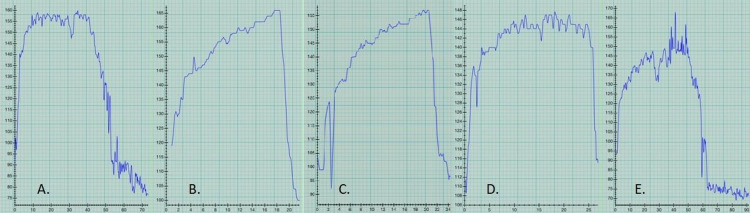
Pre-atrial fibrillation level of conditioning, as suggested by heart rate (HR) during and post-exercise. HR recorded by Series 2 I-Watch and graphed using Heart Graph application on an iPhone. A. 4/19/17 Paddle boarding (peak HR 150s), B. 6/8/18 Running (HR 160s), C. 1/21/19 Climbing/descending stairs with 45lb backpack (HR 150s), D. 3/3/19 Cycling standing up (HR 140s), E. 6/27/19 Paddle boarding (HR 150-160s); all with rapid HR recovery.

Levels of aerobic conditioning performing different exercises prior to the appearance of AF are shown in Figure 1. Heart rate (HR) was recorded with a Series 2 I-Watch and graphed using Heart Graph iPhone application. The user did not receive any financial subsidies for using these applications. For paddle boarding, HR would rapidly go up to the 150s-160s (Figure 1A: April 2017, Figure 1E: June 2019) and then recover rapidly to less than 100. Running and stair climbing with a backpack would similarly get the HR up to the 150s-160s and cycling would increase the pulse to the 140s, again with rapid recovery (Figure 1B-D). There never was any excessive shortness of breath prior to the appearance of AF.

**Figure 2:**
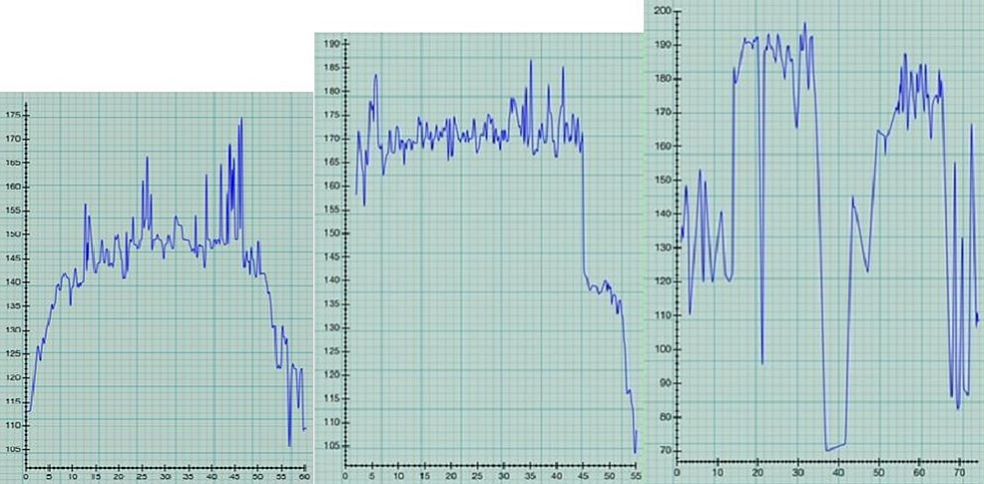
Post paddle boarding atrial fibrillation (AF) before medications. Episodes A and B were suspected in hindsight due to exaggerated fatigue (both), nausea (B), and spiking heart rates greater than 170. Episode C (heart rate 190s) was confirmed by the physician paddle boarder by taking his pulse and listening with a stethoscope, plus it was accompanied by extreme fatigue, near syncope, and severe nausea. A. 7/25/19 1st suspected AF episode, B. 8/20/19 2nd suspected AF episode, C. 9/7/19 confirmed AF.

**Figure 3:**
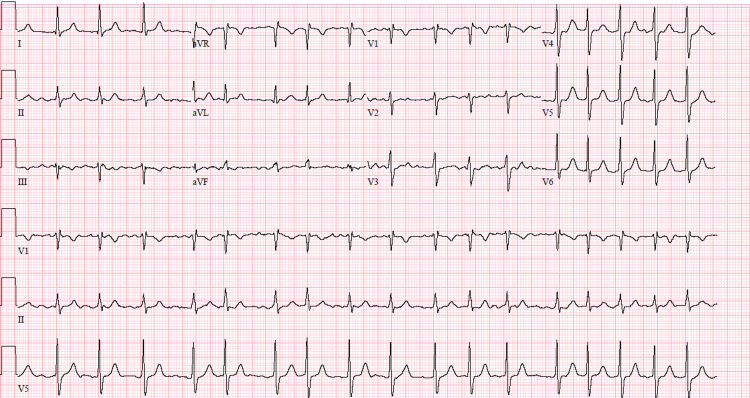
Electrocardiogram (ECG) obtained on admission showing atrial fibrillation with rapid ventricular response at rate of 107 beats per minute

The onset of a predisposition toward post paddle boarding AF likely occurred between July and September of 2019 (Figure 2). Suspected AF occurred during paddle boarding on 7/25/19 and 8/20/19 with HR spiking to greater than 170 associated with exaggerated fatigue and shortness of breath, and one time with nausea (8/20/19). The September 2019 episode was associated with a HR in the 190s, extreme fatigue, worse nausea, and near syncope, to the point that a 15-minute break was taken in order to recover and finish paddling the 3-mile circuit. During the break, the pulse was irregularly irregular, and it persisted after returning home, resulting in a hospital admission for AF with rapid ventricular response (RVR) (Figure 3).

After treatment with metoprolol, normal sinus rhythm (NSR) returned and the patient was discharged on propafenone (225mg twice daily), after consultation with an Electrophysiology (EP) Cardiologist (author JG). The hospitalization was otherwise significant for a negative cardiac stress test, normal cardiac echo (ejection fraction 50-55%, no valvular heart disease, left atrial diameter 4 cm), and normal troponins and TSH. A subsequent sleep study was negative for obstructive sleep apnea.

**Figure 4:**
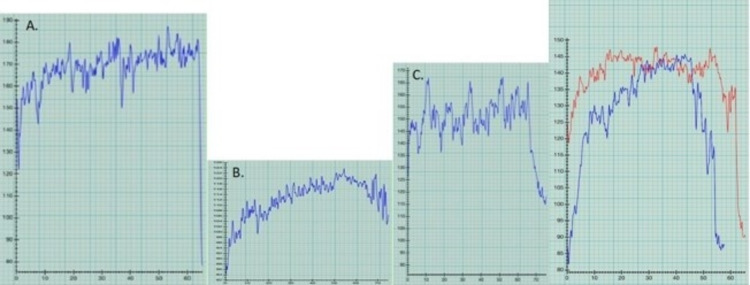
Post paddle boarding atrial fibrillation (AF) in spite of medications (graphs A, B) confirmed by MD by manual pulse heart rate, stethoscope, and single lead EKG (Kardia); lack of AF with tennis (graph C), followed by elimination of post paddle boarding AF after atrial ablation and use of low dose metoprolol (graph D). A. 9/17/19, AF (peak HR 170-180s) associated with easy paddle boarding in spite of propafenone 225 mg bid, B. 10/1/19 AF (peak HR 120s) associated with easy paddle boarding in spite of dofetilide 500 mg bid, C. 10/17/19 State championship tennis match without AF (peak HR 160s, pulse taken manually multiple times during match) on dofetilide, D. Paddle boarding without AF 3 or 4 months after atrial ablation (5/4/20) and while on metoprolol succinate 25 mg per day, 8/10/20 (red) and 9/11/20 (blue).

After discussion with the EP Cardiologist, paddle boarding was reattempted resulting in a return of AF with RVR, in spite of paddling easily and being on propafenone (Figure 4A). The patient was readmitted and started on dofetilide (500mg twice daily) and encouraged to try paddle boarding again. Dofetilide controlled the rate (less than 130), but AF returned during the outing (Figure 4B) and continued until sometime that night; normal sinus rhythm (NSR) was present upon awakening the next morning. Notably, dofetilide did keep the heart in NSR during two doubles matches played to win the South Carolina over 70 team doubles championship (Figure 4C shows the HR during one of those matches).

The patient eventually underwent atrial catheter ablation on May 4th 2020, after which he developed AF again during paddle boarding in August 2020. He was then started on low dose metoprolol succinate (25 mg daily) and resumed paddle boarding with no recurrence of AF on more than 40 different 2-mile paddle boarding outings, while still allowing near-maximum age-predicted HR (220-70 = 150; 140-150 HR achieved, Figure 3D) and no associated side effects.

## Discussion

Atrial fibrillation (AF) is the most prevalent arrhythmia in the world [1]. It has a significant public health burden, because of the associated increased risk of stroke, heart failure, mortality, and bleeding risk, if on anticoagulation. The lifetime AF risk is 25% for individuals above 40 years, and greater than 50%, if the individual has lifestyle-related risk factors, such as hypertension, elevated cholesterol, and/or heart disease.

It is important to note that the patient never developed AF or symptoms suggesting AF with any of the other exercises mentioned above, and his I-Watch was checked after each exercise episode was completed. The consistency with which AF occurred only with paddle boarding led us to search the literature for other cases of AF provoked by paddle boarding. To our knowledge, this is the first case of post-exercise AF associated only with paddle boarding.

Exercise-induced AF is an infrequent yet well-known entity, it has been described in the medical literature as a syndrome called paroxysmal AF in young and middle-aged athletes (acronym: PAFIYAMA) [1]. PAFIYAMA has been associated with cycling, cross country skiing, distance swimming, mountain walking, and middle and long-distance running [2]. Previous studies have noted that the frequency of vigorous exercise was associated with a higher risk of developing AF in joggers and younger individuals. This risk reportedly has decreased as the population’s age increased [3].

Long-term endurance exercise can cause anatomical changes in an athlete’s heart including myocardial fibrosis, especially in the atria, which can be a risk factor for AF. Other factors that might link long-term excessive exercise and AF include increased vagal and sympathetic tone, increased atrial size, inflammatory changes, and bradycardia. In roughly 20% of competitive athletes, the left atrium may be enlarged which may be a predictor for AF. It is important to note that the relationship between left atrial size and AF is more complex in athletes than in non-athletes [4].

Guideline documents recommend that PAFIYAMA be managed in consultation with an electrophysiologist [1,5-7]. Early ablation may be beneficial for athletes with impaired physical performance, especially if they want to remain competitive [5]. For some athletes whose performance was impaired by AF, multiple pulmonary vein ablations have proven successful in restoring full competitive function and allowed these athletes to participate in their activities again [6-9].

It is worth speculating about why post paddle boarding AF occurred in this aging athlete. Based on HR alone, paddle boarding produced higher sustained HR (160s) than stand up cycling, going up and downstairs with a 45 lb. backpack, running, or playing competitive tennis, suggesting it was responsible for the greatest stress on the heart. This is corroborated using the estimated maximum HR calculation 220 - age equal 150 for a 70-year-old, so heart rates in the 160s are likely to be at the high end of heart stress, even with a prior history of 50 plus years of aerobic exercise. As stated above, both increased sympathetic and parasympathetic heart stimulation can trigger AF, and this is especially true for aging athletes [10,11]. Although it is not known which of these two stimuli is more important, most experts in the field have speculated that adrenergic signals are likely dominant. During exercise, high heart rates are associated with catecholamine release which stimulates the heart by binding to cardiac surface α, β1, β2, and β3 receptors [11]. Then, through a sodium-calcium exchange mechanism, this can potentially favor the generation of AF.

The American Heart Association and American College of Cardiology both recommend the following tests be done for any athlete with AF: performance-enhancing/illicit drug use questioning, thyroid function testing, electrocardiography, and echocardiography (Class I recommendations) [7]. It has been suggested that moderate levels of exercise will have a protective effect and may even decrease the incidence of AF, whereas excessive endurance exercise heightens the risk of AF [9]. As for anticoagulation with exercise-related AF, recommendations are based on the presence of known risk factors for thromboembolism [12]. Individuals who participate in sports with a higher risk of trauma should be excluded. Our patient was anticoagulated from the first documented episode until two months after his catheter ablation [13]. Treating modifiable risk factors should be an essential cornerstone of care along with medications/interventions [14].

## Conclusions

In summary, a 69-year-old athlete developing AF only after paddle boarding. Two antiarrhythmic drugs alone (propafenone, dofetilide) were unsuccessful. Atrial ablation and low-dose metoprolol prevented any further paddle board-associated episodes of AF. The fact that metoprolol was successful likely means that for this athlete, increased sympathetic tone was the dominant trigger. Further research needs to be done to know whether this approach has value for the treatment of paroxysmal AF in other athletes.
